# Attentive 3D-Ghost Module for Dynamic Hand Gesture Recognition with Positive Knowledge Transfer

**DOI:** 10.1155/2021/5044916

**Published:** 2021-11-18

**Authors:** Jinghua Li, Runze Liu, Dehui Kong, Shaofan Wang, Lichun Wang, Baocai Yin, Ronghua Gao

**Affiliations:** ^1^Beijing Key Laboratory of Multimedia and Intelligent Software Technology, Faculty of Information Technology, Beijing University of Technology, Beijing 100124, China; ^2^Beijing Research Center for Information Technology, Agriculture, Beijing 100097, China

## Abstract

Hand gesture recognition is a challenging topic in the field of computer vision. Multimodal hand gesture recognition based on RGB-D is with higher accuracy than that of only RGB or depth. It is not difficult to conclude that the gain originates from the complementary information existing in the two modalities. However, in reality, multimodal data are not always easy to acquire simultaneously, while unimodal RGB or depth hand gesture data are more general. Therefore, one hand gesture system is expected, in which only unimordal RGB or Depth data is supported for testing, while multimodal RGB-D data is available for training so as to attain the complementary information. Fortunately, a kind of method via multimodal training and unimodal testing has been proposed. However, unimodal feature representation and cross-modality transfer still need to be further improved. To this end, this paper proposes a new 3D-Ghost and Spatial Attention Inflated 3D ConvNet (3DGSAI) to extract high-quality features for each modality. The baseline of 3DGSAI network is Inflated 3D ConvNet (I3D), and two main improvements are proposed. One is 3D-Ghost module, and the other is the spatial attention mechanism. The 3D-Ghost module can extract richer features for hand gesture representation, and the spatial attention mechanism makes the network pay more attention to hand region. This paper also proposes an adaptive parameter for positive knowledge transfer, which ensures that the transfer always occurs from the strong modality network to the weak one. Extensive experiments on SKIG, VIVA, and NVGesture datasets demonstrate that our method is competitive with the state of the art. Especially, the performance of our method reaches 97.87% on the SKIG dataset using only RGB, which is the current best result.

## 1. Introduction

Hand gesture is one of the most natural interaction ways, and hand gesture recognition based on video aims to attain the symbol describing hand gesture action automatically. Hand gesture recognition has been widely applied to human-computer interaction [1], automatic vehicle, virtual reality [2], or augmented reality. However, achieving high-accuracy dynamic hand gesture recognition still faces many challenges since of the variable illuminations, complex background and different individuals etc. As is known to us, RGB data is sensitive to the illumination, while depth data is the opposite. That is, multimodal data such as RGB-D contains many complementary information between modalities, which is helpful to improve hand gesture recognition performance [3, 4]. Therefore, most state-of-the-art hand gesture recognition methods use multimodal data, which offer significant improvements than those by only one modality. However, in reality, RGB and depth are not always available at the same time.

One expected approach is to utilize RGB-D multimodal data to train model so as to receive more knowledge, while in the test stage, hand gesture can be recognized based on the well-trained multimodal model with only one kind of modality information. To this end, Abavisani et al. [5] proposed a MTUT model, which is a feasible approach for the above idea, in which I3D network is selected as the baseline representing each modality [6], and minimizing semantic loss is proposed to implement the cross-modality knowledge transfer. However, there still exist two problems. Firstly, the spatial-temporal features of dynamic hand gesture for each modality are not fully described. Secondly, the knowledge is only transferred unidirectionally without considering which modality contains more and better knowledge.

This paper aims to improve the aforementioned two problems. With regard to feature representation, Han et al. [7] proposed a kind of Ghost module, which is integrated to 2D-CNN architecture and has attained the improved representation performance. It is well known to us that the traditional CNN extracts more abundant features by leveraging more convolutional kernels and commonly leads to some ghost results, that is, some feature maps are similar. To address this problem, GhostNet sacrifices some convolutional filters, which uses cheaper linear operations to replace these convolution operators and generates richer feature maps. Motivated by 2D GhostNet, this paper proposes a 3D-Ghost module for 3D video description, which reduces the redundancy existing in 3D-CNN feature maps and attains more abundant features. In addition, attention mechanism has been successfully applied to all kinds of computer vision tasks [8]; therefore, we design a kind of spatial attention mechanism to pay more attention to the hand area under variable conditions. We combined the 3D-Ghost and spatial attention to the baseline I3D, and named the novel network as 3DGSAI. The 3DGSAI network is used to extract the features of each modality separately, i.e., the 3DGSAI does not share parameters. They are trained independently so that dynamic hand gestures can be recognized in the subsequent testing stage based on only one kind of modality data.

With regard to knowledge transfer, Abavisani et al. [5] suppose that depth data has better classification performance than RGB data. Therefore, the knowledge is unidirectionally transferred from depth channel to RGB by minimizing spatiotemporal semantic alignment (SSA) loss. To avoid negative transfer, when the classification accuracy of depth is lower than that of RGB, knowledge transfer is forbidden. As we know, in most cases, depth is better than RGB for hand gesture classification. However, this is not available for any data. The ideal solution is that the knowledge is adaptively transferred according to the classification performance of each modality. For this reason, instead of transferring only from depth to RGB channel, in this paper, we propose an adaptive transfer parameter, which determines the direction of knowledge transfer by the difference of two-modality classification loss. In summary, the main contributions of this paper are as follows:

- We propose a novel three-dimensional convolutional neural network 3DGSAI for dynamic hand gesture classification in which 3D-Ghost module and spatial attention mechanism are proposed and integrated to the baseline I3D network; therefore, more abundant and key features for each modality are attained.
- We design an adaptive parameter for positive knowledge transfer, which guarantees that the knowledge always transfers from strong modality to weak one. The transferring sources depend on modality with lower classification loss. The proposed cross-modal positive transfer method enhanced the classification performance of the weak modality and cannot reduce that of the strong modality.

The rest of this paper is organized as follows.  Section 2 introduces the related work.  Section 3 describes the proposed method.  Section 4 presents the experiments and results. Finally, we conclude this paper.

## 2. Related Work

### 2.1. Dynamic Hand Gesture Recognition

Recently, many deep learning methods have been applied to dynamic hand gesture recognition in an end-to-end manner [9, 10]. Unlike image classification task, dynamic hand gesture recognition, a task for the video, needs to consider not only the spatial information of each frame in a video sequence but also the temporal correlation between frames, which brings great challenges to hand gesture recognition task.

According to the number of the input modalities, the methods of hand gesture recognition can be divided into two kinds, i.e., single-modality recognition and multimodality recognition [11]. Among them, unimodal hand gesture recognition only uses one type of modality information, which is more convenient and simpler but necessarily lacks the assistance from other modalities. Therefore, recent hand gesture recognition methods are integrating multimodal information to improve recognition accuracy. Various multimodal hand gesture recognition methods based on 3D-CNN and RNN have been proposed in the literature. In [12], a 3D-CNN was proposed to fuse data streams from multiple sensors to recognize dynamic hand gestures. A 3D-CNN-based method was proposed in [13], which combines normalized depth and image gradient values to complete the classification of dynamic hand gestures. Reference [14] adopted C3D network to extract the features of RGB modality and depth, respectively, and then the concatenated features are input to SVM classifier. The work in [15] used a C3D network and ConvLSTM for feature extraction from each modality data stream (RGB and Depth). Reference [16] explored the role of attention mechanism in LSTM and introduced a new LSTM variant to effectively complete the recognition task. Reference [17] presented a spatiotemporal attention-based 3D-CNN to capture high-quality features to classify multimodal dynamic gestures. Although multimodal gesture recognition is competitive in recognition accuracy, it also has certain limitations, that is, the data acquisition conditions are relatively harsh. In actual application scenarios, if there is only one kind of modality data, the multimodal framework cannot work. Therefore, in this work, we train multiple unimodal classification networks synchronously based on multimodal gesture data, that is, we embed multimodal information into each unimodal network so as to achieve high-efficiency unimodal gesture recognition.

### 2.2. Feature Representation

Feature representation is important for any classification task. Hand gesture is a kind of dynamic action performed by hand with spatial and temporal characteristics. Hand feature representation is quite important for hand gesture recognition and also faces many challenges. As we know, hand gestures contain plentiful semantics. The amplitudes of hand gesture action are local and detailed and affected by many factors such as illumination and background. Zhang et al. [18] proposed to use 3D-CNN + ConvLSTM to learn 2D spatiotemporal feature maps to obtain the local spatial information and global temporal information and then further use 2D-CNN to obtain high-level spatiotemporal feature from the 2D feature maps. A feature representation method based on pose attention mechanism was proposed in [19] which is used in video action recognition. In [20], a two-stream consensus-voting network (2SCVN) including temporal stream network and spatial stream network was proposed to obtain final spatiotemporal feature representation that indicates hand motion by consensus voting. Reference [21] adopted a temporal feature representation method and proposed multikernel temporal block (MKTB) and global refinement block (GRB) by modeling time series and combining the two blocks to effectively explore the spatiotemporal feature representation of hand gestures. The feature representation of the proposed framework is based on I3D network, and we proposed 3DGSAI network, which aims to obtain more effective features to realize high-performance single-modality gesture recognition.

### 2.3. Transfer Learning

Supervised deep learning tends to require tremendous amount of training data, while data annotation is time consuming and laborious. Transfer learning provides a novel way to analyze data with few annotation by transferring the knowledge of the source domain to the target domain, which has achieved better transferring performance [22, 23]. Recently, transfer learning has been proven effective in many application scenarios [24, 25]. Our method aims to recognize hand gesture with only one modality while training with multimodal data. That is, multimodal knowledge is mutually learned during the training stage so as to enhance the recognition performance of single modality during the testing stage. In our method, minimizing SSA loss is used to realize knowledge transfer, especially the adaptive parameter is proposed to control the positive knowledge transfer.

## 3. Methodology

In this section, we describe the proposed methods in detail. The dynamic hand gesture recognition task of this paper is defined as follows: recognizing dynamic hand gesture only by unimodal RGB data {*x*_*i*_^*m*^, *y*} or Depth ones {*x*_*i*_^*n*^, *y*} when testing, but to levegage multimodal knowledge to improve the unimodal recognition accuracy, multimodal RGB-D hand gesture video sequence {*x*_*i*_^*m*^, *x*_*i*_^*n*^, *y*_*i*_} are both used for training. The method in this paper is not limited to the two modalities of RGB and depth and also can be extended to more modalities. We make two efforts to improve the baseline hand gesture recognition performance. On the one hand, each modality can individually learn their own expressive representation so as to attain good classification accuracy; on the other hand, cross-modal positive knowledge transferring is necessary. To this end, 3DGSAI network is proposed to extract more expressive features for each modality, and adaptive positive knowledge transfer is proposed to enhance the weak modality network. In the following, this paper introduces the details one by one.

### 3.1. Overall Framework

**Figure 1:**
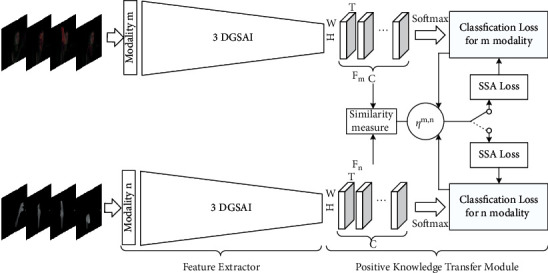
Overview of our hand gesture recognition framework.

As shown in Figure 1, our dynamic hand gesture recognition framework can be divided into two parts: feature extractor and positive knowledge transfer module. With the input RGB and depth video clip sequence, feature extractor uses the proposed 3DGSAI network to obtain the spatiotemporal feature representation of hand gestures individually, while positive knowledge transfer module is implemented by enforcing the similarity measure loss to the modality with lower classification accuracy, which is penalized by an adaptive parameter *η*^*m*,*n*^.

In this architecture, the baseline of the 3DGSAI network is I3D. To achieve more expressive features, the proposed 3D-Ghost module and spatial attention mechanism are integrated to the baseline network. The 3D-Ghost module makes the network get richer and more flexible feature maps, which include a large amount of abundant semantic knowledge. Since the spatial attention mechanism may enforce the network to pay more attention to the region of interest [26], we designed spatial attention module in the 3DGSAI network to focus on the arm and hand joints. In summary, our proposed 3DGSAI network can obtain high-quality hand feature representations for dynamic hand gesture recognition.

In view of the different advantages of the color modality and the depth modality in describing different actions, for some gestures, the color modality is more expressive, while for other gestures, the depth is more discriminative, so the weight parameters of 3DGSAI network in the two modalities are not shared. However, this paper aims to enhance the weak modality by learning knowledge from the strong one. Therefore, the positive knowledge transfer module is proposed. Here, *F*_*m*_ and *F*_*n*_ are, respectively, the output features of modality *m* and *n* extracted by 3DGSAI networks, and the similarity of *F*_*m*_ and *F*_*n*_ is measured by SSA loss. In fact, measuring and minimizing the similarity between two-modality feature maps can be seen as a process of transferring knowledge. In order to ensure that knowledge is transferred from one modality with excellent performance to the other with poor performance in any training steps, we propose a positive knowledge transfer parameter in the SSA loss to control the direction of knowledge transfer. During training, the two-modality networks are trained with their own loss functions, and the SSA loss is added to the classification loss of the weak modality to ensure the positive transfer of knowledge. Here, the classification loss uses the cross-entropy loss function.

### 3.2. Feature Extractor

#### 3.2.1. 3D-Ghost Module

GhostNet is a newly proposed simple and effective classification network, in which the Ghost module is based on the following observation and analysis.

**Figure 2:**
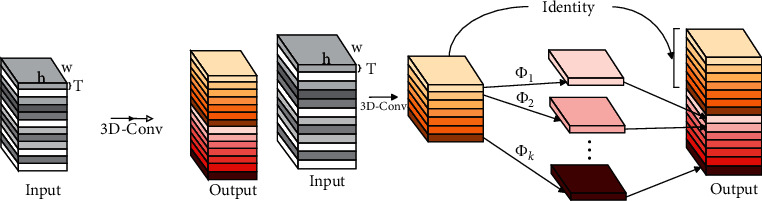
Traditional 3D convolution and proposed 3D-Ghost module. (a) The 3D convolution layer. (b) The 3D-Ghost module.

The feature maps of CNN often contain redundant information, that is, there is a ghost phenomenon, and this redundant information may be the key to excellent network performance and can be obtained through simpler linear calculations. However, Ghost module is only used in 2D-CNN. In this paper, to classify dynamic hand gesture video sequence, 3D-Ghost module is proposed and integrated into the 3D convolutional neural network. The 3D-Ghost module can be used as a plug-and-play component to upgrade the existing 3D convolutional neural network.  Figure 2 shows the comparison between the traditional 3D convolution operation and the 3D-Ghost module. It can be seen that many convolutional filters are used to extract feature maps in the traditional 3D convolutional layer, while in the 3D-Ghost module, less convolutional filters are used to generate a set of feature maps, and then a series of linear operations Φ_1_, Φ_2_,…, Φ_*k*_ acts on these feature maps to generate a large number of 3D ghost feature maps.

In practical application, given the input data *X* ∈ *ℝ*^*C*×*T*×*h*×*w*^, where *C* represents the number of input channels, *T* represents the number of frames in the input video, and *h* and *w* are the height and width of the input data, respectively, the operation of 3D convolution to generate feature maps can be expressed as

(1) $$\begin{array}{c} Y=X*f+b, \end{array}$$

where *∗* is the 3D convolution operation, *b* is the bias term, *Y* ∈ *R*^*T*′×*h*′×*w*′×*N*^ is the output feature map with *N* channels, and *f* ∈ *R*^*C*×*k*×*k*×*k*×*N*^ is the 3D convolution kernel in this layer. Moreover, *T*′, *h*′, and *w*′ are the frames, height, and width of the output data, and *k* × *k* × *k* is the size of convolution kernel *f*.

The above is a conventional 3D convolution operation, and the output feature maps contain more redundancy than 2D convolution operation, and some of them may be similar to each other. Therefore, we believe that it is not necessary to use a large number of FLOPs and parameters to generate these redundant 3D feature maps and propose 3D-Ghost module to replace the 3D convolution operation. To be specific, we first use one 3D convolution to generate *M* inherent feature maps *Y*′ ∈ *R*^*T*′×*h*′×*w*′×*M*^.

(2) $$\begin{array}{c} Y^{\prime }=X*f^{\prime } \end{array}$$

where *f*′ ∈ *R*^*C*×*k*×*k*×*k*×*M*^ represents the utilized filters, *M* ≤ *N*, and the bias term is omitted for simplicity. The hyperparameters such as filter size, stride, and padding are the same as those in the conventional 3D convolution operation (equation (1)) to keep the spatiotemporal dimensions (i.e., *T*′, *h*′, and *w*′) of the output feature map consistent. In order to further obtain the required *N* feature maps, we apply a series of 3D linear operations to each 3D inherent feature map in *Y*′ according to the following formula to generate *S* 3D ghost feature maps.

(3) $$\begin{array}{c} y_{i,j}=\Phi_{i,j}\left(y_{i}^{\prime }\right),\;\forall i=1,\ldots ,M,j=1,\ldots ,S, \end{array}$$

where *y*_*i*_′ is the *i*-th 3D inherent feature map in *Y*′ and Φ_*i*,*j*_ represents the *j*-th (except the last) 3D linear operation, which is used to generate the *j*-th 3D ghost feature map *y*_*ij*_. In other words, *y*_*i*_′ may have one or more 3D ghost feature maps {*y*_*ij*_}_*j*=1_^*S*^. The last Φ_*i*,*S*_ represents the inherent mapping, which is used to retain the 3D inherent feature maps. Through formula (3), *N*=*M* · *S*, a total of *N* 3D feature maps *Y*=[*y*_11_, *y*_12_,…, *y*_MS_] can be obtained as the output data of the 3D-Ghost module, as shown in Figure 2(b).

In general, the working mechanism of the 3D-Ghost module is to first use a 3D convolution operation to generate a set of inherent feature maps. Then, these inherent feature maps are subjected to a 3D convolution operation to generate ghost feature maps, while retaining the inherent feature maps of the first convolution. Finally, the inherent feature maps and ghost feature maps are connected in series to replace the original 3D convolution operation. The proposed 3DGSAI network integrates the 3D-Ghost module to obtain richer and more flexible feature map representation. Since the correlation matrix is obtained by multiplying the normalized feature map and its transpose, the feature map is richer, and the semantic information contained in the correlation matrix will be richer.

**Figure 3:**
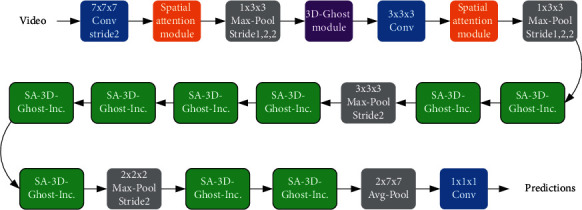
The overall structure of the 3DGSAI network.

**Figure 4:**
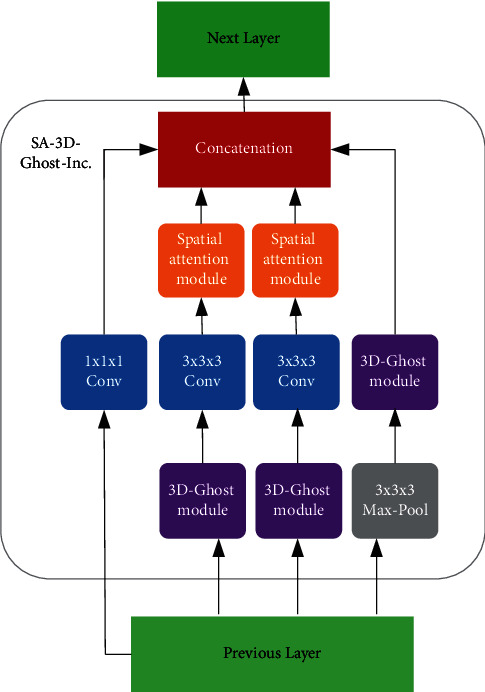
Architecture of SA-3D-Ghost-Inception submodule.

The whole framework proposed in this paper is a two-stream collaborative learning structure, and adding the 3D-Ghost module will transfer high-quality semantic knowledge between the two channels to improve the performance of the model. As shown in Figure 3, for the structure of the proposed 3DGSAI network, the 3D-Ghost module and the spatial attention mechanism defined in Section 3.2.2 are integrated on the basis of I3D. The specific structure of the SA-3D-Ghost-Inception submodule is shown in Figure 4.

### 3.3. Spatial Attention Mechanism

The attention mechanism is essentially to extract key information from a lot of information and ignore other unimportant information to improve the performance of the model. To make the classification network pay more attention to the features of hands and arms, we integrated the spatial attention module to the proposed 3DGSAI network, which is helpful to extract the key features of the input RGB and depth video sequences.

The core of the attention mechanism is to learn the weight parameter. First, the importance of the semantic information represented by each element on the feature map is learned. Then, each element on the feature map is assigned weight according to its importance degree. The greater the weight, the higher the importance degree. For a feature map *F*  ∈  *ℝ*^*C*×*T*×*h*×*w*^, an attention map *M*(*F*) ∈ *ℝ*^*C*×*T*×*h*×*w*^ can be obtained through the attention mechanism module, where *M*(*F*) is the weight used to characterize the importance of the feature. After that, *M*(*F*) and the original feature map *F* are multiplied element by element to extract the key features in each frame of image sequences, which forms the attention.

The computation of weight coefficient *M*(*F*) can be formulated as formula (4), where *σ* is the sigmoid function, *f*^*d*×*d*×*d*^ corresponds to the 3D convolutional layer with *d*  = 7 and *d*  = 3 respectively, and *F*_avg_ and *F*_max_ are the channel descriptions of average pooling and max pooling, respectively. The input is a feature map *F* of *C* × *T* × *h* × *w*, and the average pooling and maximum pooling of a channel dimension are performed, respectively, to obtain two *T* × *h* × *w* × 1 channel descriptions, and these two descriptions are spliced together according to the channel. After passing through the 7 × 7 × 7 or 3 × 3 × 3 convolutional layer and the activation function sigmoid, the weight coefficient *M*(*F*) is obtained.

(4) $$\begin{array}{c} M\left(F\right)=\sigma \left(f^{d\times d\times d}\left(\left[\text{Avgpool}\left(F\right),\text{Maxplool}\left(F\right)\right]\right)\right)\\ =\sigma \left(f^{d\times d\times d}\left[F_{\text{avg}};F_{\max}\right]\right). \end{array}$$

Finally, the original feature map *F* and the weight coefficient *M*(*F*) are multiplied element by element to obtain a new feature *F*^*∗*^ weighted by attention. As shown in formula (5), where ⊗ represents the elementwise multiplication.

(5) $$\begin{array}{c} F^{*}=F⊗M\left(F\right). \end{array}$$

### 3.4. Positive Knowledge Transfer Module

The proposed framework adopts a collaborative learning method, which encourages the weak classification modality to obtain the knowledge of the strong classification modality. In the training phase, when a modality network cannot learn discriminative representation, the knowledge of another modality network can be used to improve its representation. Repeated occurrences of this situation will result in a better representation of the network in a collaborative manner.

The baseline method realizes the transfer of knowledge from a perfect modality to another modality by constraining the semantic consistency of the two modalities:

(6) $$\begin{array}{c} \ell_{\text{SSA}}^{m,n}=\rho^{m,n}{\left\|\text{corr}\left(F_{m}\right)-\text{corr}\left(F_{n}\right)\right\|}_{F}^{2}. \end{array}$$

Here, corr(*F*_*m*_) and corr(*F*_*n*_), respectively, represent the correlation matrix of the output features of the *m*-th and *n*-th channels. *ρ*^*m*,*n*^ is the focus regularization parameter, which is used to control the direction of knowledge transfer and force the better-performing network to transfer knowledge to the poorer-performing network. The focus regularization parameter of the baseline network is

(7) $$\begin{array}{c} \rho^{m,n}=S\left(e^{\beta \Delta \ell }-1\right)=\left\{\begin{array}{cc} e^{\beta \Delta \ell }-1, & \Delta \ell >0,\\ 0, & \Delta \ell \leq 0. \end{array}\right. \end{array}$$

Among them, Δ*ℓ*=*l*_cls_^*m*^ − *l*_cls_^*n*^, Δ*ℓ* represents the difference between the classification loss of *m* and *n* channels, and *l*_cls_^*m*^ and *l*_cls_^*n*^ represent the classification loss corresponding to the *m*-th modality network and the *n*-th modality network, respectively. A positive value of Δ*ℓ* indicates that the performance of network *n* is better than that of network *m*. At this moment, when training network *m*, it is hoped that *ρ*^*m*,*n*^ is a large value to force network *m* to simulate the representation of network *n*.

However, there is a problem with the baseline method. That is, the defaults are that the feature representation of one modality network (depth) is stronger than that of the other modality network (color). The focus regularization parameter of the baseline only makes the depth modality network transfer knowledge to the color modality, which is only a one-way knowledge transfer process. However, we found that in different datasets or different training epochs, it is not certain that the feature map representation of one modality must be better than the other modality. Therefore, in response to this problem, we proposed a new adaptive parameter for positive knowledge transfer *η*^*m*,*n*^, so that the two-modality 3DGSAI networks can be self-adaptively learned in both directions, and Δ*ℓ* is also multiplied in front of the exponent to speed up the convergence of the network:

(8) $$\begin{array}{c} \eta^{m,n}=\left\{\begin{array}{cc} \left(\ell_{\text{cls}}^{m}-\ell_{\text{cls}}^{n}\right)e^{\beta \Delta \ell }-1, & \Delta \ell >0,\\ \left(\ell_{\text{cls}}^{n}-\ell_{\text{cls}}^{m}\right)e^{-\beta \Delta \ell }-1, & \Delta \ell \leq 0. \end{array}\right. \end{array}$$

The proposed adaptive parameter *η*^*m*,*n*^ for positive knowledge transfer can be used to distinguish which modality network has higher discriminability. The adaptivity is determined according to positive or negative Δ*ℓ* during the training process. The optional definition of *η*^*m*,*n*^ ensures the cross-modality positive and effective transfer of knowledge. Hence, the mutual learning between the two modalities greatly enhances the collaboration of the whole framework. The improved SSA loss used in this paper is as follows:

(9) $$\begin{array}{c} \ell_{\text{SSA}}^{m,n}=\eta^{m,n}{\left\|\text{corr}\left(F_{m}\right)-\text{corr}\left(F_{n}\right)\right\|}_{F}^{2}. \end{array}$$

### 3.5. Full Objective of the Cross-Modality Networks

This paper aims to obtain knowledge from cross-modality learning during training, while in the testing stage, the recognition task is implemented by more flexible way, i.e., hand gesture is recognized under more general conditions. For instance, only RGB or depth data are provided. To this end, each modality of multimodalities must be trained individually, that is, each modality has their respective input flows and loss functions. With regard to the mutual learning of cross-modality knowledge, this paper adds the SSA loss to weaker modality. That is, for each epoch, two modalities are trained by their own classification loss. In addition, the modality with higher classification loss will be constrained by SSA loss. Especially, this process is dynamic and adaptive depending on the difference value of two-modality classification loss. SSA is computed according to equation (9). In summary, the loss function of each modality can be summarized as follows.

When Δ*ℓ* > 0, the loss functions of channel *m* and channel *n* are

(10) $$\begin{array}{c} \ell_{m}=\ell_{\text{cls}}^{m}+\lambda \ell_{\text{SSA}}^{m,n},\\ \ell_{n}=\ell_{\text{cls}}^{n}. \end{array}$$

When Δ*ℓ*≤ 0, the loss functions of channel *m* and channel *n* are

(11) $$\begin{array}{c} \ell_{m}=\ell_{\text{cls}}^{m},\\ \ell_{n}=\ell_{\text{cls}}^{n}+\lambda \ell_{\text{SSA}}^{m,n}, \end{array}$$

where *λ*  = 0.05 is a trade-off hyperparameter. The proposed framework encourages each modality network to improve the feature representation through knowledge transfer in the training phase. The training procedure for our proposed framework is presented in Algorithm 1.

During the testing phase, for any given modality data, the corresponding single-modality network is executed. It means that once the whole two-modality framework is trained, each modality network can independently complete high-precision unimodal hand gesture recognition based on its corresponding input modality stream.

## 4. Experiment Results and Analysis

In this section, the experiments are evaluated from two facets. The first experiment aims to verify the recognition accuracy of the proposed method relative to the state-of-the-art methods on three public hand gesture datasets, and the second one is to verify the influence of each component in our framework through ablation experiment.

### 4.1. Dataset and Experimental Setup

The experiments are evaluated on three public datasets, including Sheffield Kinect Gesture (SKIG) dataset [27], VIVA hand gesture dataset [28], and NVGesture dataset [29]. All experiments use multimodal data for training and only provide unimodal data during testing.

SKIG is a multimodal dynamic hand gesture dataset, which contains 1080 RGB-D video sequences of 10 hand gesture classes. All hand gesture samples are performed by 6 subjects using 3 different palm shapes (fist, flat, and index only) under 2 illumination conditions (highlight and low one) and 3 backgrounds (white paper, wood texture, and newspaper). The dataset is divided into three subsets according to the subjects: subject1 + subject2, subject3 + subject4, and subject5 + subject6. 3-fold cross-validation is used to evaluate the proposed framework.

The VIVA hand gesture dataset is a multimodal dynamic hand gesture dataset for real-world driving scenes, which mainly includes complex backgrounds, unstable lighting, and obstacle occlusion. The dataset is captured using a Microsoft Kinect device and contains 885 visible RGB-D video sequences from 8 subjects with 19 hand gesture classes. Since this dataset is not divided into training set and test set, 8-fold cross-validation is used to evaluate the performance of gesture recognition according to subjects.

The NVGesture dataset is collected with multiple sensors and multiple viewpoints for studying the human-computer interface. It contains 1532 hand gesture videos, which were recorded by 20 subjects in a car simulator with lighting conditions, and a total of 25 classes of hand gestures. The hand gestures are recorded with SoftKinetic DS325 device as the RGB-D sensor and DUO-3D for the infrared streams. In the experiment of this article, the RGB modality data and depth modality data of this dataset are used.

All the experiments are performed on Ubuntu 18.04 with a GeForce GTX 3090 GPU. Our proposed framework is implemented using PyTorch 1.7 [30]. We use Adam optimizer, a stochastic gradient descent algorithm, to train the whole model.

In all experiments, *λ* is set to 50 × 10^−3^, *β*=2, and the learning rate is set to 0.0001. In the training stage, batch size is set to 2, that is, two 64-frame RGB-D video sequences are sent to the model in each iteration. Our method consists of two stages, pretraining for each modality 3DGSAI classification network and positive knowledge transfer between the two modalities. Therefore, epoch times are, respectively, set to 60 for pretraining and 15 epochs for knowledge transfer.

With regard to the choice of comparison methods, we mainly follow two principals. One is to choose the most advanced methods in the field of dynamic hand gesture recognition, such as I3D, C3D, and MTUT, which are all used as comparison methods on three datasets. The other is to choose the classic and representative methods reported on each used dataset, which are different from dataset to dataset. Therefore, different comparison methods are employed to evaluate performance on different datasets.

### 4.2. Evaluation of Recognition Performance

This section aims to evaluate the recognition performance of the proposed method objectively. The experiments are conducted on the SKIG, VIVA, and NVGesture dataset. For each dataset, we report the recognition accuracy of the proposed method and comparison ones. All comparative experiments in this paper are reproduced based on the experimental settings in the previous section.

#### 4.2.1. SKIG Dataset

In this section, we analyze the experimental results on the SKIG dataset. The SKIG dataset is one of the widely used datasets for hand gesture recognition, which contains many common and important hand gesture actions used in daily life. This paper selects some advanced and classic hand gesture recognition methods for comparison, which includes domain-adaptive method for leaning discriminative spatiotemporal features, which is optimized by restricted graph-based genetic programming (RGGP) [27], the method of jointly encoding local and global information for single depth sequence (depth context) [31], and four advanced deep learning methods (MRNN [32], I3D [6], 3D-CNN + CLSTM [15], and MTUT [5]). All the results are reported by averaging the classification accuracy over 3-fold cross-subject cross-validation.

Table 1 shows the performance of the dynamic hand gesture recognition methods tested on the RGB and depth modalities of the SKIG dataset. As can be seen from the table, the performance of deep learning methods is significantly better than that of the traditional machine learning method RGGP. The performance of depth context is general and only for depth sequences, which has certain limitations in the field of multimodal hand gesture recognition. It is easy to see that C3D + CLSTM performs perfect, especially the accuracy for depth modality achieves 98.7%. Compared with C3D + CLSTM, the performance of our proposed method on RGB modality is improved by 1.94%, and the performance of depth modality is equivalent to C3D + CLSTM. With regard to the baseline network MTUT, the proposed framework has a distinct improvement in both RGB and depth modality. It is worth mentioning that our proposed method achieves 97.87% for RGB modality on the SKIG dataset, which is currently the best recognition performance with only RGB modality.

#### 4.2.2. VIVA Dataset

The VIVA dataset is a difficult and challenging dataset, and the data are captured in a vehicle with varied background, illumination, and subjects. Especially, the trajectories and shapes difference of some hand gestures are very subtle although they belong to different categories, which further increases the difficulty of hand gesture recognition. We compare our method with classical deep learning methods for video action classification and hand-crafted HOG + HOG2 feature method.  Table 2 shows the testing accuracy of single modality with different hand gesture recognition methods. HOG + HOG2 is a hand-crafted feature extracting approach [28], and CNN:LRN is a recurrent 2D-CNN-based method [13]. As can be seen, the 3D-CNN-based methods, C3D [33], I3D [6], and MTUT [5], are better than the 2D-CNN-based and hand-crafted method. We also can see that MTUT and our proposed method are better than those methods in which both training and testing only use one modality. Especially, our method is 1.47% and 1.18% higher than the MTUT method in the RGB domain and depth domain, respectively. The experimental results implied that our proposed 3DGSAI network and cross-modality positive knowledge transfer ensured the expressive feature extracting, and thus the performance of classification has exceeded MTUT.

**Figure 5:**
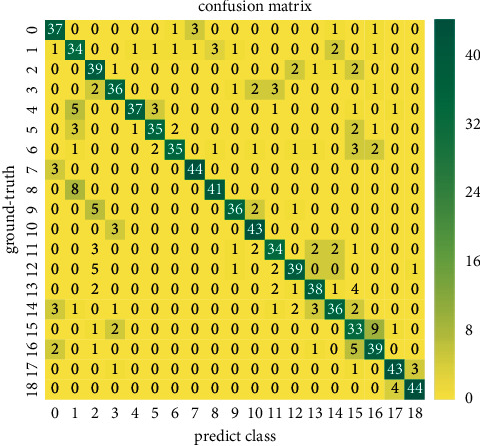
Confusion matrix of RGB modality classification via 3DGSAI trained on the VIVA dataset.

**Figure 6:**
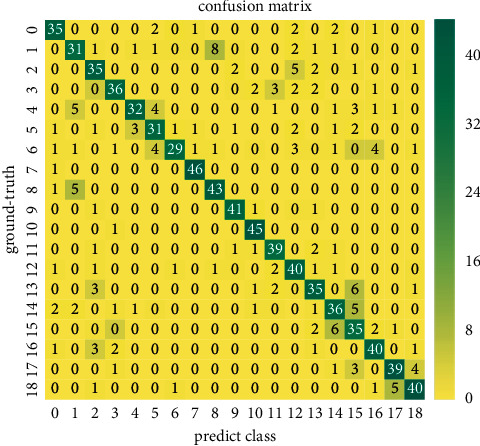
Confusion matrix of depth modality classification via 3DGSAI trained on the VIVA dataset.

Figures 5 and 6 show the confusion matrix recognized via RGB or depth individually. It can be seen that our method is less confusing in most categories and presents a diagonal confusion matrix. However, because some hand gesture categories only differ in details, there are still some gestures that cannot be correctly classified. For example, since category 1 (Swipe L) and category 8 (Scroll L) have similar movement trajectory features at the beginning of the hand gesture action, there is a certain degree of confusion. In addition, similar pose features of category 15 (Rotate CCW) and category 16 (Rotate CW) cause 3DGSAI network classification errors.

#### 4.2.3. NVGesture Dataset

Table 3 lists the comparison results of our method and the recent state-of-the-art methods: HOG + HOG2 [28], two-stream CNNs [34], improved dense trajectories(iDT) [35], C3D [33], I3D [6], and MTUT [5]. The iDT method is generally regarded as the best hand-crafted method with the highest hand gesture recognition rate. However, similar to the previous experiments, we observe that the performance of hand gesture recognition based on 3D-CNN is better than that of CNN and other hand-crafted methods, and our methods have achieved the best performance. From Table 3, we also see that the recognition accuracy based on depth modality is better than that of RGB, which is most likely due to the perfect data quality of depth video; instead, RGB video is with more noise, and hence the performance of the depth modality is stronger than that of the RGB modality. From this table, we can conclude that our work not only extracts high-quality features but also enables the network to develop stronger feature representation capabilities in a collaborative manner through the two-way knowledge transfer, which improves the learning process of the whole framework.

### 4.3. Ablation Experiment

The improvement of the proposed method is reflected in the 3DGSAI network and adaptive parameter *η*^*m*,*n*^ for positive knowledge transfer, so in the following we analyze their influence on hand gesture recognition accuracy, respectively.

All ablation experiments are performed on the SKIG dataset. The default parameter settings are *λ*=50 × 10^−3^, *β*=2.

#### 4.3.1. Influence of 3DGSAI Network

The baseline network of this paper is I3D classification network. Relative to I3D, the improvement of 3DGSAI network mainly lies in proposing a 3D-Ghost module and introducing a kind of spatial attention mechanism. Therefore, we evaluate the gains of these two points and the overall 3DGSAI network on three datasets, respectively, and the results are shown in Tables 4–6. It is not difficult to see that only introducing 3D-Ghost module or spatial attention is helpful for improving the recognition accuracy of the baseline, especially the gain of 3D-Ghost module is bigger. As we can see, the spatial attention module can improve the recognition rate in both RGB domain and depth domain by about 0.35%, while the 3D-Ghost module can achieve performance improvement of about 0.7% in both the RGB modality and the depth under this framework. We also can see that when 3D-Ghost module and spatial attention are introduced to the baseline together, we get the 3DGSAI network, which achieved the best recognition accuracy, and the gain is about 1.15%. The experimental results imply that the 3D-Ghost module and spatial attention in 3DGSAI have contributed to extracting expressive features for classification.

In summary, the above experiments have proved that the 3D-Ghost module can improve accuracy and, at the same time, has fewer 3D convolution kernels than traditional 3D convolutions, that is, fewer parameters. Therefore, the 3D-Ghost module also has advantages in terms of model time efficiency. We conducted experiments on the size of weight, FLOPs, and number of parameters of the baseline I3D network and I3D + 3D-Ghost module on the VIVA dataset. The position of the added 3D-Ghost module is shown in Figures 3 and 4, but the spatial attention module is removed. The experimental results are shown in Table 7. It can be concluded that adding the 3D-Ghost module to the baseline I3D network made the model faster.

#### 4.3.2. Influence of Adaptive Parameter

This section aims to evaluate the effect of the proposed adaptive parameter *η*^*m*,*n*^ for positive knowledge transfer, and the baseline is the original focal regularization parameter *ρ*^*m*,*n*^. To this end, we conducted two experiments. One is the baseline+3DGSAI, and the other is baseline+3DGSAI+*η*^*m*,*n*^. According to Tables 8–10, the adaptive parameter has improved the classification accuracy by about 0.6%. The distinct improvement confirmed that the proposed adaptive parameter *η*^*m*,*n*^ for positive knowledge transfer is indeed essential for hand gesture recognition. This adaptive parameter *η*^*m*,*n*^ can judge the pros and cons of the feature representation based on the difference between the loss of the two modal networks and force the network with high classification accuracy to transfer knowledge to the network with low classification accuracy. In other words, the adaptive parameter *η*^*m*,*n*^ can maintain the positive transfer of knowledge during the whole training process.

### 4.4. Effect of Unimodal Improvements on Multimodal Fusion

As mentioned above, the experimental results have proved that unimodal classification accuracy has been improved by 3DGSAI and cross-modal positive knowledge transfer, which verified the effectiveness of the proposed method. As known to us, theoretically, multimodal fusion necessarily produces better results, and the work of this paper is also applied to multimodal fusion. That is to say, when RGB and depth video data are both provided for testing, this work is feasible, especially the proposed unimodal improvements definitely increase the performance of multimodal fusion. In this section, we adopt the decision level fusion to evaluate the effect of unimodal improvements on multimodal fusion. Since the recognition accuracy of unimodality of SKIG dataset has been pretty high, we only conduct the multimodal fusion experiments on the VIVA and NVGesture datasets. In Tables 11 and 12, we compare our method with the state-of-the-art multimodal hand gesture recognition methods on the VIVA dataset and the NVGesture dataset, respectively. As can be seen from the tables, our method achieves the highest recognition accuracy on these two datasets. It is not difficult to see that the recognition accuracy of multimodal fusion is better than that of only single modality shown in Tables 3 and 5. We also can see that the recognition accuracy of multimodality fusion with knowledge transfer between the modalities is better than that without cross-modal transfer. Especially, the multimodal fusion based on the proposed unimodal improvements performs better than that of the baseline MTUT.

## 5. Conclusion

In summary, the proposed method of this paper consists of two facets. To attain more expressive hand gesture features for classification task, we proposed a new 3DGSAI network in which a kind of 3D-Ghost module is proposed to extract more expressive and richer features, and at the same time, spatial attention mechanism is introduced to enable the learning model to pay more attention to the hand region, which strengthens the high-quality feature extraction. To ensure the positive knowledge transfer occurring in RGB modality and depth one, this paper proposed an adaptive parameter for positive knowledge transfer, which ensured that the knowledge transferred from the strong modality to the weak one throughout the whole training process. Since the adopted framework leverages multimodality data to train and a single modality for testing, our method has great potential for real-world applications. Extensive experiments on three public datasets demonstrate that our proposed method is competitive or superior to related works.

## Figures and Tables

**Algorithm 1:**
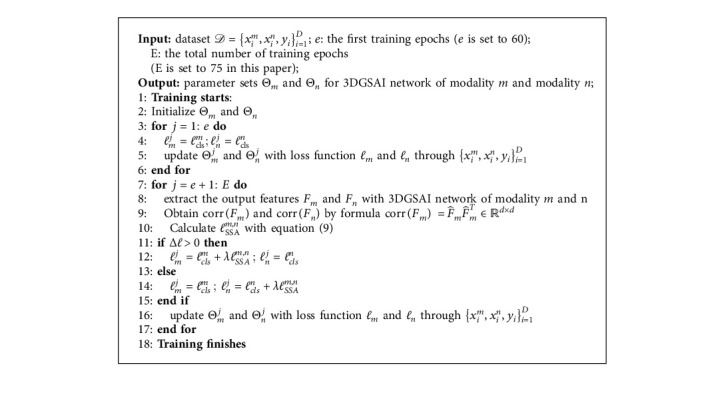
Training process of the whole framework

**Table 1:** 3-fold cross-subject average accuracy (%) of different hand gesture methods on the SKIG dataset.

Method	Testing modality	
	RGB	Depth
RGGP [27]	84.60	76.10
Depth context [31]	—	95.37
MRNN [32]	91.60	95.90
I3D [6]	95.74	96.58
C3D + CLSTM [15]	95.93	98.70
MTUT [5]	95.93	96.85
**Ours**	**97.87**	**98.70**

In the experiment results, the best recognition accuracy is in bold.

**Table 2:** 8-fold cross-subject average accuracy (%) of different hand gesture methods on the VIVA dataset.

	Testing modality	
Method	RGB	Depth
HOG + HOG2 [28]	52.3	58.6
CNN:LRN [13]	57.0	65.0
C3D [33]	71.26	68.32
I3D [6]	77.84	74.46
MTUT [5]	80.03	78.97
**Ours**	**81.50**	**80.15**

In the experiment results, the best recognition accuracy is in bold.

**Table 3:** Accuracy (%) of different gesture recognition methods on the NVGesture dataset.

	Testing modality	
Method	RGB	Depth
HOG + HOG2 [28]	24.5	36.3
Two-stream CNNs [34]	54.6	—
iDT [35]	59.1	—
C3D [33]	69.3	78.8
I3D [6]	70.54	82.57
MTUT [5]	71.58	84.02
**Ours**	**73.03**	**85.48**

In the experiment results, the best recognition accuracy is in bold.

**Table 4:** The influence of different components of 3DGSAI network on SKIG dataset.

	Testing modality	
Method	RGB	Depth
Baseline	95.93	96.85
Baseline + SA	96.30	97.31
Baseline + 3D-Ghost	96.67	97.78
**Baseline** **+** **3DGSAI**	**97.22**	**98.24**

In the experiment results, the best recognition accuracy is in bold.

**Table 5:** The influence of different components of 3DGSAI network on VIVA dataset.

	Testing modality	
Method	RGB	Depth
Baseline	80.03	78.97
Baseline + SA	80.32	79.30
Baseline + 3D-Ghost	80.66	79.53
**Baseline** **+** **3DGSAI**	**80.99**	**79.75**

In the experiment results, the best recognition accuracy is in bold.

**Table 6:** The influence of different components of 3DGSAI network on NVGesture dataset.

	Testing modality	
Method	RGB	Depth
Baseline	71.58	84.02
Baseline + SA	71.78	84.44
Baseline + 3D-Ghost	72.20	84.65
**Baseline** **+** **3DGSAI**	**72.41**	**84.85**

In the experiment results, the best recognition accuracy is in bold.

**Table 7:** Computational complexity comparison of the I3D network and its improved version with the 3D-Ghost module on VIVA dataset.

Model	Weights (M)	FLOPs (G)	Parameters (M)
I3D [6]	48	111.42	12.31
**I3D** **+** **3D-Ghost**	**45**	**108.16**	**11.23**

In the experiment results, the best recognition accuracy is in bold.

**Table 8:** The effect of adaptive parameter *η*^*m*,*n*^ on the recognition accuracy (%) of SKIG dataset.

Method	Testing modality	
	RGB	Depth
Baseline + 3DGSAI	97.22	98.24
**Baseline+3DGSAI+** *η* ^**m**,**n**^	**97.87**	**98.70**

In the experiment results, the best recognition accuracy is in bold.

**Table 9:** The effect of adaptive parameter *η*^*m*,*n*^ on the recognition accuracy (%) of VIVA dataset.

Method	Testing modality	
	RGB	Depth
Baseline + 3DGSAI	80.99	79.75
**Baseline** **+** **3DGSAI** **+** *η*^**m**,**n**^	**81.50**	**80.15**

In the experiment results, the best recognition accuracy is in bold.

**Table 10:** The effect of adaptive parameter *η*^*m*,*n*^ on the recognition accuracy (%) of NVGesture dataset.

Method	Testing modality	
	RGB	Depth
Baseline + 3DGSAI	72.41	84.85
**Baseline** **+** **3DGSAI** **+** *η*^**m**,**n**^	**73.03**	**85.48**

In the experiment results, the best recognition accuracy is in bold.

**Table 11:** Accuracy comparison of different multimodal fusion-based hand gesture recognition methods on the VIVA dataset.

Method	Fused modalities	Accuracy
HOG + HOG2 [28]	RGB + depth	64.5
CNN:LRN [13]	RGB + depth	74.4
CNN:LRN:HRN [13]	RGB + depth	77.5
C3D [33]	RGB + depth	77.4
I3D [6]	RGB + depth	83.10
MTUT [5]	RGB + depth	86.08
**Ours**	RGB + depth	**86.97**

In the experiment results, the best recognition accuracy is in bold.

**Table 12:** Accuracy comparison of different multimodal fusion-based hand gesture recognition methods on the NVGesture dataset.

Method	Fused modalities	Accuracy
HOG + HOG2 [28]	RGB + depth	36.9
I3D [6]	RGB + depth	83.82
MTUT [5]	RGB + depth	85.48
**Ours**	RGB + depth	**86.72**

In the experiment results, the best recognition accuracy is in bold.

## Data Availability

The SKIG dataset used to support the findings of this study is available from the corresponding author upon request. VIVA dataset can be accessed from the following link: http://cvrr.ucsd.edu/LISA/hand.html. NVGesture dataset can be accessed from the following link: https://research.nvidia.com/publication/online-detection-and-classification-dynamic-hand-gestures-recurrent-3d-convolutional.

## References

[1] Rautaray S. S., Agrawal A. (2015). Vision based hand gesture recognition for human computer interaction: a survey. *Artificial Intelligence Review*.

[2] Lv Z., Halawani A., Feng S., ur Rehman S., Li H. (2015). Touch-less interactive augmented reality game on vision-based wearable device. *Personal and Ubiquitous Computing*.

[3] Miao Q., Li Y., Ouyang W. Multimodal gesture recognition based on the resc3d network.

[4] Wang H., Wang P., Song Z., Li W. Large-scale multimodal gesture recognition using heterogeneous networks.

[5] Abavisani M., Vaezi Joze H. R., Patel V. M. Improving the performance of unimodal dynamic hand-gesture recognition with multimodal training.

[6] Carreira J., Zisserman A. Quo vadis, action recognition? a new model and the kinetics dataset.

[7] Han K., Wang Y., Qi T., Guo J., Xu C., Xu C. GhostNet: more features from cheap operations.

[8] Xu K., Jimmy B., Ryan K. Show, attend and tell: neural image caption generation with visual attention.

[9] Adegun A. A., Viriri S., Ogundokun R. O. (2021). Deep learning approach for medical image analysis. *Computational Intelligence and Neuroscience*.

[10] Chen G., Ge K. (2020). A fusion recognition method based on multifeature hidden markov model for dynamic hand gesture. *Computational Intelligence and Neuroscience*.

[11] Tran T.-H., Tran H.-N., Doan H.-G. (2019). *Dynamic Hand Gesture Recognition from Multi-Modal Streams Using Deep Neural Network*.

[12] Molchanov P., Gupta S., Kim K., Pulli K. Multi-sensor system for driver’s hand-gesture recognition.

[13] Molchanov P., Gupta S., Kim K., Kautz J. Hand gesture recognition with 3d convolutional neural networks.

[14] Li Y., Miao Q., Tian K. Large-scale gesture recognition with a fusion of rgb-d data based on the c3d model.

[15] Zhu G., Zhang L., Shen P., Song J. (2017). Multimodal gesture recognition using 3-D convolution and convolutional LSTM. *IEEE Access*.

[16] Zhang L., Zhu G., Mei L. Attention in convolutional LSTM for gesture recognition.

[17] Huang J., Zhou W., Li H. (2019). Attention-based 3D-CNNs for large-vocabulary sign language recognition. *IEEE Transactions on Circuits and Systems for Video Technology*.

[18] Liang Z., Zhu G., Shen P. Learning spatiotemporal features using 3DCNN and convolutional LSTM for gesture recognition.

[19] Du W., Wang Y., Yu Q. RPAN: an end-to-end recurrent pose-attention network for action recognition in videos.

[20] Duan J., Wan J., Zhou S., Guo X., Li S., Li Z. (2018). A unified framework for multi-modal isolated gesture recognition. *ACM Transactions on Multimedia Computing, Communications, and Applications*.

[21] Yi Y., Ni F., Ma Y. High performance gesture recognition via effective and efficient temporal modeling.

[22] Pan S. J., Yang Q. (2010). A survey on transfer learning. *IEEE Transactions on Knowledge and Data Engineering*.

[23] Torrey L., Shavlik J. (2010). Transfer learning. *Handbook of Research on Machine Learning Applications and Trends: Algorithms, Methods, and Techniques*.

[24] Oquab M., Leon B., Laptev I., Sivic J. Learning and transferring mid-level image representations using convolutional neural networks.

[25] Perera P., Patel V. Deep transfer learning for multiple class novelty detection.

[26] Woo S., Park J., Lee J.-Y., Kweon I. S. CBAM: convolutional block Attention module.

[27] Li L., Ling S. Learning discriminative representations from RGB-D video data.

[28] Ohn-Bar E., Trivedi M. M. (2014). Hand gesture recognition in real time for automotive interfaces: a multimodal vision-based approach and evaluations. *IEEE Transactions on Intelligent Transportation Systems*.

[29] Molchanov P., Yang X., Gupta S., Kim K., Stephen T., Kautz J. Online detection and classification of dynamic hand gestures with recurrent 3D convolutional neural networks.

[30] Paszke A., Gross S., Chintala S. Automatic differentiation in pytorch.

[31] Liu M., Liu H. (2016). Depth Context: a new descriptor for human activity recognition by using sole depth sequences. *Neurocomputing*.

[32] Nishida N., Nakayama H. Multimodal gesture recognition using multi-stream recurrent neural network.

[33] Tran Du, Bourdev L., Fergus R., Torresani L., Paluri M. Learning spatiotemporal features with 3d convolutional networks.

[34] Simonyan K., Zisserman A. Two-stream convolutional networks for action recognition in videos.

[35] Wang H., Oneata D., Verbeek J., Schmid C. (2016). A robust and efficient video representation for action recognition. *International Journal of Computer Vision*.

